# Increased de novo DNA Methylation Enzymes in Sperm of Individuals with Varicocele

**DOI:** 10.22074/cellj.2021.7265

**Published:** 2021-08-29

**Authors:** Moloud Rashidi, Marziyeh Tavalaee, Homayon Abbasi, Michail Nomikos, Mohammad Hossein Nasr-Esfahani

**Affiliations:** 1. Department of Animal Biotechnology, Reproductive Biomedicine Research Center, Royan Institute for Biotechnology, ACECR, Isfahan, Iran; 2. Department of Biology, Faculty of Science, NourDanesh Institute of Higher Education, Isfahan, Iran; 3. Isfahan Fertility and Infertility Center, Isfahan, Iran; 4. College of Medicine, Member of QU Health, Qatar University, Doha, Qatar

**Keywords:** DNA Methylation, DNMT1, DNMT3A, DNMT3B, Varicocele

## Abstract

**Objective:**

Chronic genital heat-stress associated with varicocele leads to DNA hypo-methylation of spermatozoa. The objective of this study was comparing level of DNA methyl-transferases (DNMTs) in sperm of men suffering varicocele with fertile individuals.

**Materials and Methods:**

In this case-control study, semen samples were obtained from 35 infertile men with varicocele (grade II or III) and 26 fertile men. Sperm parameters were assessed according to World Health Organization (WHO) protocol. DNMTs enzymes level were assessed by flow cytometer and fluorescence microscope. mRNAs expression of these DNMTs were also assessed by real-time reverse transcription polymerase chain reaction (RT-PCR).

**Results:**

DNMT1 and DNMT3A proteins were mainly localized in equatorial and mid-piece regions of sperm head, respectively, while DNMT3B protein appeared to be localized mainly in equatorial and anterior regions of sperm head. In contrast to DNMT1, expression and percentage of DNMT3A and DNMT3B at RNA and protein levels were significantly higher in the varicocele group compared to the fertile group (P<0.05). In addition, significant correlations were found between sperm concentration and motility as well as DNMT1 and DNMT3B proteins levels in the infertile individuals with varicocele (P<0.05). Additionally, significant correlations were observed between abnormal sperm morphology with DNMTs proteins in the infertile individuals with varicocele.

**Conclusion:**

Unlike DNMT1, which is involved in maintenance of DNA methylation at both RNA and protein levels, expression of de novo methylation enzymes (DNMT3A and DNMT3B) at both levels were increased in the varicocele group compared to the fertile group. Based on literature, this increase might be due to the dual roles played by DNMT3A and DNMT3B, as methyl-transferases in normal condition as well as dehydroxymethylases in stress condition, like varicocele. Although, this hypothesis needs further validation.

## Introduction

It is currently well-established that epigenetic phenomena play a central role in developmental biology. Epigenetic inheritance and germline reprogramming are considered as two sides of the same coin and epigenetic annotations of chromatin including DNA methylation, as well as acetylation, ubiquitination and methylation of histones are believed to be the main executive marks of epigenesis (1). Among these marks, DNA methylation mainly occurs in CpG islands located in gene promoters and typically acts to repress gene transcription. In addition, other important roles are considered for DNA methylation, such as chromatin condensation and nuclear organization, particularly at regions of constitutive heterochromatin (2). DNA methyltransferases (DNMTs) constitute a family of enzymes that catalyze the transfering a methyl group to DNA. Until now, according to literature, three main active DNA methyltransferases (DNMT1, DNMT3A and DNMT3B) and one DNA methyltransferase without catalytic activity (DNMT3L) are present in cells (3).

DNMT1 is responsible for preservation of methylation pattern through methyltransferase activity during cell division (4). Unlike DNMT1, the other two methyltransferases (DNMT3A and DNMT3B) play a central role in development. Knocked out-mice studies showed that disruption of any of these two active DNA methyltransferase genes was lethal and each of these two Dnmts had its specific targets. With regard to *Dnmt3L*, the respective knocked out mice are viable but their male offsprings are sterile and do not produce mature sperms, while their female offsprings are fertile, but fail to deliver viable pups. This difference has been mainly related to the fact that *Dnmt3L* lacks catalytic activity and functions as a co-factor for *Dnmt3A* and *Dnmt3B* (5, 6). 

As stated above, activity of DNMT3A and DNMT3B is indispensable for spermatogenesis. In this regard, developmental studies in mice revealed that primordial germ cells (PGC) showed different degrees of DNA methylation during the course of their development to gametes; they become completely de-methylated during gonadal formation and differentiation and gradually regain their methylation status by the time they reach spermatogonia and spermatocyte stages (7). In contrast to mice, methylation status of PGC, spermatogonia and spermatocytes are not yet well-characterized in human. Following the erasure of methylation, progressive methylation is established by the aid of DNMT3A, DNMT3B and DNMT3L (8). Considering the pivotal role of DNA methylation in development and its correlation with large numbers of diseases/disorders (9), several studies investigated the relationship of DNA methylation level, male fertility and pregnancy outcome (10-15). 

One of the major etiology of male infertility is varicocele. In this condition, an abnormal enlargement of the pampiniform venous plexus in the scrotum results in disturbed testicular temperature, hypoxia and backflow of toxicants to testis, all of which can alter epigenetic status of cells within testis (16). Recent studies revealed that incidence of varicocele in men with primary infertility (21-41%) and men with secondary infertility (75-81%) are high (17). To explore the exact pathophysiology of varicocele, researchers focused on different aspects of this phenomenon (18). Previous studies showed that both percentage and intensity of sperm DNA methylation by flow cytometry method were significantly lower in infertile individuals with varicocele, compared to fertile individuals, possibly due to hyperthermia (19, 20). Considering the fact that epigenetic status is influenced by environmental changes especially stressors (21), we aimed to access expression of DNMT1, DNMT3A and DNMT3B at RNA and protein levels in sperm of individuals with varicocele, compared to fertile individuals.

## Materials and Methods

For this case-control study, we received Ethics Committee approval from Royan Research Institute, Iran (IR.ACECR.ROYAN.REC.1394.38). All the individuals provided written informed consent for their participation and usage of their semen samples, information and/or data. The study was performed on sperm from semen samples of 35 infertile men with varicocele (grade II or III) and 26 fertile individuals who referred for family balancing, as control group. The male ages were ranged from 27 to 43 years with a mean of 34.95 ± 4.58 in fertile individuals, and from 26 to 44 years with a mean of 32.98 ± 4.04 in infertile men with varicocele. 

### Inclusion criteria

Infertile men with primary infertility, grade II or III varicocele and below the 45 years of age were included in this study. Fertile men with at least one child, below 45 year of age, candidate for preimplantation genetic diagnosis (PGD) or egg donation was also included in this study.

### Exclusion criteria

Semen samples with higher than 1 million somatic cell per ml to minimize DNA and somatic cell contamination, and men with grade I varicocele were excluded from this study.

### Sperm samples

Semen samples were obtained through masturbation from men with varicocele and fertile individuals referring to the Isfahan Fertility and Infertility Centre (Isfahan, Iran). The sexual abstinence periods were ranged from 3 to 7 days. For liquefaction, the samples were kept at room temperature in andrology laboratory for 30-60 minutes before evaluation.

Semen analysis was carried out by an experienced laboratory personnel according to World Health Organization (WHO) guidelines. We assessed sperm concentration and motility by Computer Assisted Semen Analysis (CASA; Video Test, ltd: version Sperm 2.1© 1990-2004, Russia) system, while sperm morphology was assessed after Papanicolaou staining (22).

### Flow cytometry and immunostaining techniques

**Fig.1:**
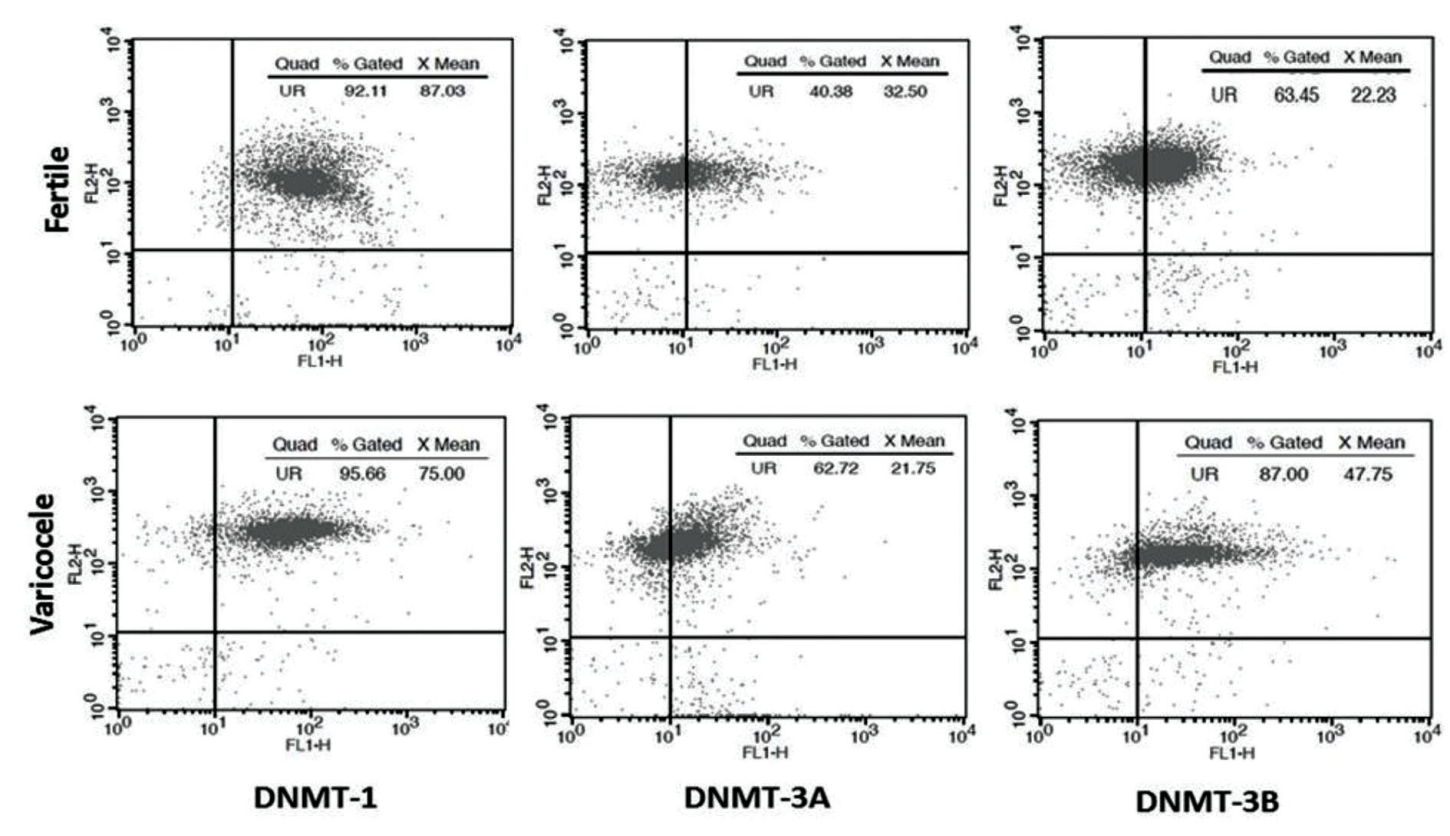
Schematic representation of flow cytometry scatter plot (DNMTs) between semen sample of a fertile man and an infertile man with varicocele. The results of DNMTs was expressed as percentage (% gated), and relative fluorescence intensity (X mean). DNMTs; DNA methyl-transferases.

**Fig.2:**
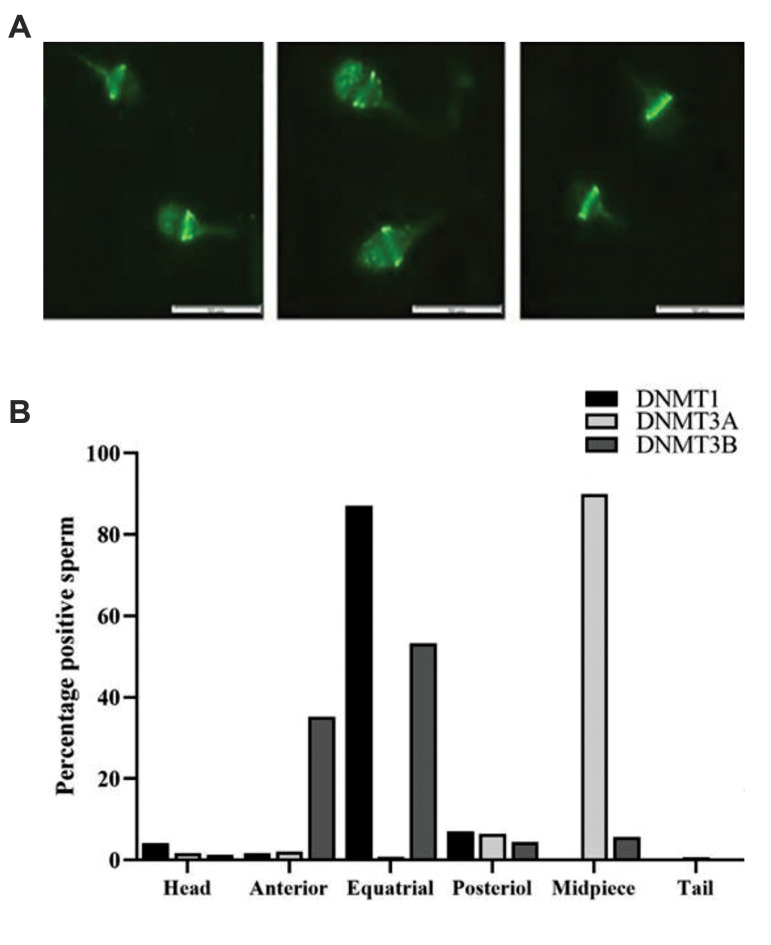
Localization of DNMTs in human sperm method. **A.** Localization of DNMTs protein in different regions of sperm. **B. **Percentage of localization of DNMTs in different regions of sperm. (scale bar: 50 µm). DNMTs; DNA methyl-transferases.

Semen samples were washed with phosphate buffered saline (PBS) and spermatozoa were fixed in 4% paraformaldehyde (Sigma, USA) in PBS for 60 minutes at room temperature. Then, the samples were washed twice with cold PBS and incubated for 10 minutes with 0.25% Triton-X-100 (Merck, Germany) in PBS for permeabilization. After two rounds of wash with PBS, the samples were incubated with 1% bovine serum albumin (BSA, Sigma, USA) in PBST [0.5% Tween-20 (Merck, Germany) in PBS] for 30 minutes to block unspecific binding. The samples were then incubated overnight at 4˚C with primary monoclonal mouse antibodies (one antibody per slide) against DNMT1, DNMT3A and DNMT3B (all from IMGENEX, USA) in 1% BSA. After two rounds of washing with PBS, the samples were incubated with FITC secondary antibody [Goat Anti-Mouse IgG Antibody; Chemicon, USA] in 1% BSA in PBST, for 1 hour at 37˚C in dark. Next, the samples were washed and counterstained with propidium iodide (PI) to distinguish derbies from sperm (23). Percentages of DNMT1, DNMT3A and DNMT3B positive sperm were detected by flow cytometer (Becton Dickinson, USA). At least 10,000 sperms were counted in each sample. Flow cytometry results of DNMTs were expressed as “percentage of positive sperm" and “relative fluorescence intensity of stained sperm" (Fig .1). Fluorescence intensity showed average color emission from cell population in the fluorescence channel. Similar staining procedure was used for detection of DNMTs localization using a fluorescence microscope (Olympus, Japan) with the appropriate filters (460-470 nm) at ×100 magnification (Fig .2A).

### Quantitative/real-time reverse transcription polymerase chain reaction

Sperm of varicocele and fertile individuals were rinsed with PBS and total RNA was extracted using Trizol (Ambion, Canada). The procedure for RNA extraction and cDNA synthesis were according to the previous study (24). To eliminate DNA contamination, RNA-containing samples were treated with DNase I (Fermentas, Burlington). Real-time specific primer pairs were designed by the Beacon designer 7.5 (PREMIER Biosoft; USA) and synthesized by Pishgam Company (Iran). The primers were:

GAPDH: F: 5´-CCACTCCTCCACCTTTGACG-3´
  R: 5´- CCACCACCCTGTTGCTGTAG-3´
DNMT1: F: 5´-AACAGAACAAGAATCGCATC-3´
  R: 5´-GGAATAACAGAGACACAGT-3´
DNMT3A: F: 5´-CCTTCTTCTGGCTCTTTG-3´
  R: 5´-GACACTTCTTTGGCATCA-3´
DNMT3B: F5-´TAGGAGAGGAGTGTGAAG-3´
  R: 5´-AAGATGAGAAATGAGGGTAG- 3´

Real-time polymerase chain reaction (PCR) was carried out according to the manufacturer’s protocol (TaKaRa, Japan) and data were analyzed with 2^-ΔΔCt ^method. Data in Real-time PCR software were plotted as cycle threshold (C_t_) values. The C_t_ of targeted mRNA (*DNMT1, DNMT3A *and *DNMT3B*) was normalized to the C_t_ of reference gene (GAPDH), and subsequently ∆C_t_ of each sample was calculated. Next, ∆C_t_ of the sample from each varicocele individual was normalized to the mean ∆C_t_ of fertile group and data was expressed as "relative expression of genes or "2^-ΔΔCt^". 

### Primers specifications

*DNMT1*: The forward and reverse primers for *DNMT1*, recognized all four variants of *DNMT1 *RNA with unique product size of 127 bp.

*DNMT3A*: The forward and reverse primers for *DNMT3A*, recognized four out of seven variants of *DNMT3A* with unique product size of 109 bp. Two other variants have four exons and if expressed, they would produce truncated protein. Furthermore, NGS-analysis from Gene Expression Omnibus (GEO): GSE69434 showed that expression of these two variants was at base line noise. The other variant was a non-coding RNA which should not be considered for assessing *DNMT3A* expression. 

*DNMT3B*: The forward and reverse primers for *DNMT3B*, recognized all six variants of *DNMT3B* RNA with unique product size of 128 bp.

### Statistical analysis

For data analysis, we used package for the Social Studies (SPSS software version 22, Inc., Chicago, IL, USA). In addition, Microsoft Word and Microsoft Excel software were used for drawing tables and figures, respectively. Kolmogorov-Smirnov test was used to assess normal distribution. We used independent samples t test to compare mean values between fertile and varicocele groups. Two-tailed Pearson correlation test was used to assess correlations between the parameters. Data were presented as mean ± standard error of mean (SEM). P<0.05 was considered statistically significant.

## Results

### Comparison of sperm parameters and male age between fertile and varicocele groups

Mean values of semen parameters of fertile men and individuals with varicocele were compared. Sperm concentration (109.71 ± 17.3 vs. 47.91 ± 6.5, P=0.006), total sperm count per ejaculate (551.32 ± 58.1 vs. 192.31 ± 54.3, P<0.001), percentage of sperm motility (47.74 ± 4.8 vs. 42.16 ± 4.3, P=0.03), percentage of abnormal morphology (94.21 ± 1.00 vs. 97.82 ± 0.3, P=0.04) and semen volume (5.73 ± 0.4 vs. 3.28 ± 0.3, P<0.001) were significantly different between the two groups. The mean values of age were 34.95 ± 4.58 and 32.98 ± 4.04 years in fertile individuals and infertile men with varicocele, respectively, suggesting no significant difference between these two groups (P=0.4). 

### Comparison of percentage and relative intensity of DNMTs between fertile and varicocele groups

**Fig.3:**
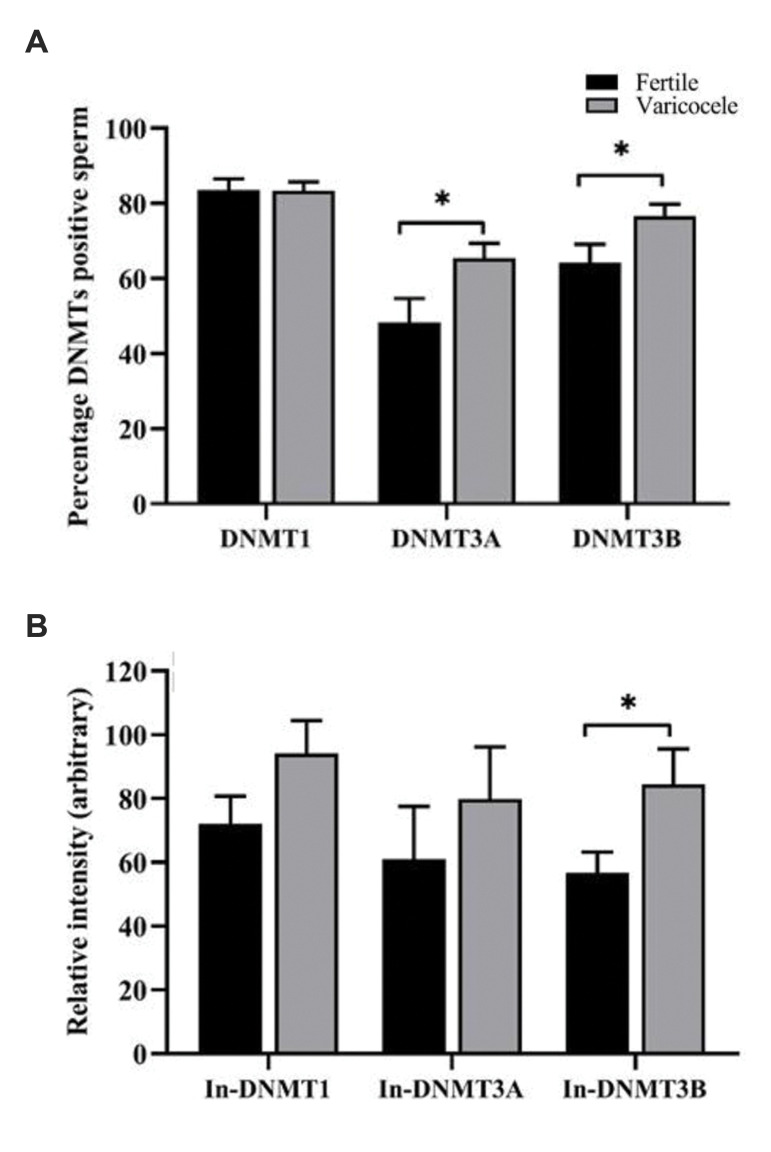
Comparison of percentage and intensity of DNMTs between fertile and varicocele groups. Comparison of **A.** Percentage and **B. **Relative fluorescence intensity (In) of DNMTs proteins between fertile men and infertile men with varicocele. Independent-samples t test was used for comparison of values between varicocele and fertile groups. DNMT; DNA methyl-transferases and *; Shows significant difference between two groups at Data were presented as mean ± standard error of mean (SEM). P<0.05 was considered statistically significant.

The percentage of DNMTs positive sperm were compared between fertile men and individuals with varicocele (Fig .3A). Percentage of DNMT1 positive sperm were similar between the two groups (fertile: 83.51 ± 3.00 vs. varicocele: 83.41 ± 2.3, P=0.97). However, percentage of DNMT3A (fertile: 48.34 ± 6.35 vs. varicocele, 65.42 ± 3.9, P=0.02) and DNMT3B positive sperm (fertile: 64.31 ± 4.8 vs. varicocele: 76.61 ± 3.1, P=0.03) were significantly higher in varicocele compared to fertile group.

Relative fluorescence intensities of DNMTs, assessed by flow cytometry, were compared between fertile men and individuals with varicocele (Fig .3B). The relative fluorescence intensities of both DNMT1 (fertile: 72.03 ± 8.00 vs. varicocele: 94.14 ± 10.3, P=0.11) and DMNT3A (fertile: 61.01 ± 16.5 vs. varicocele: 79.8 ± 16.3, P=0.43) were similar between the two groups. However, relative fluorescence intensity of DNMT3B (fertile: 56.75 ± 6.4 vs. varicocele: 84.40 ± 11.1, P=0.03) was significantly higher in varicocele group, compared to fertile group.

**Table 1:** Correlations between percentages of DNMT-positive spermatozoa and sperm parameters. The data was analyzed with two-tailed Pearson correlation test


Semen parameters	DNMTs groups	DNMT1 (%)	DNMT3A (%)	DNMT3B (%)
		Pearson correlation (r)		

Sperm concentration (10^6^/ml)	Total population	0.28^*^	-0.06	-0.04
	Fertile	0.29	-0.01	-0.01
	Varicocele	0.39^*^	0.31	0.34^*^
Total sperm count (10^6^/ejaculate)	Total population	0.38^**^	0.13	0.06
	Fertile	0.41	0.29	0.26
	Varicocele	0.34	0.38^*^	0.29
Sperm motility (%)	Total population	0.51^**^	0.17	0.09
	Fertile	0.52^**^	0.26	-0.19
	Varicocele	0.49^**^	0.18	0.47^**^
Abnormal sperm morphology (%)	Total population	-0.04	-0.13	0.09
	Fertile	-0.06	-0.43^*^	0.00
	Varicocele	-0.41^*^	-0.39^*^	-0.46^**^


DNMT; DNA methyl-transferases, *; Correlation is significant at the 0.05 level (two-tailed), and **; Correlation is significant at the 0.01 level (two-tailed).

### Comparison of relative expression of DNMTs at mRNA level between fertile and varicocele groups

**Fig.4:**
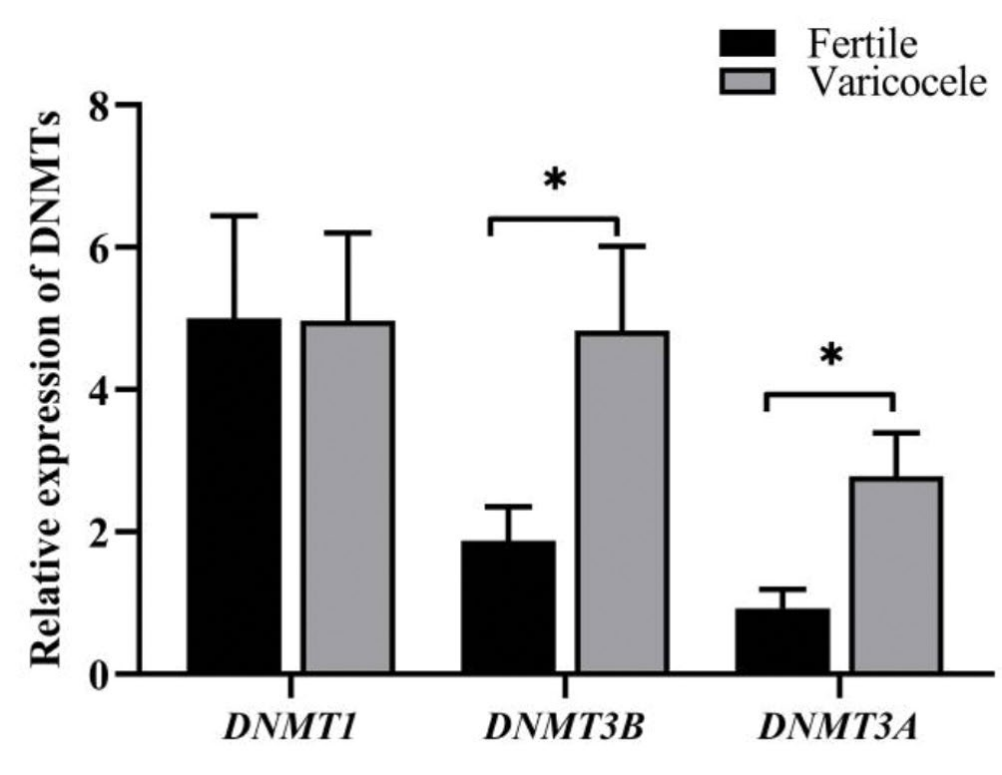
Comparison of relative expression of DNMTs at mRNA level between fertile men and infertile men with varicocele. Independent-samples t test was used for comparison of values between varicocele and fertile groups. DNMT; DNA methyl-transferases and *; Shows significant difference between two groups at P<0.05.

In contrast to relative expression of *DNMT1* (fertile: 5.00 ± 1.4 vs. varicocele: 4.97 ± 1.2, P=0.98), the relative expression of both *DNMT3A* (fertile: 1.87 ± 0.5 vs. varicocele: 4.83 ± 1.2; P=0.02) and *DNMT3B* (fertile: 0.93 ± 0.26 vs. varicocele: 2.78 ± 0.61, P=0.008) were significantly higher in individuals with varicocele in comparison with fertile men (Fig .4). 

### Correlations between percentage of DNMTs protein with sperm parameters

Correlation between sperm DNMTs proteins with semen parameters were assessed in fertile, varicocele and total population groups (Table 1). Among the analyzed correlations, positive significant correlations were observed between sperm concentration with percentage of sperm DNMT1 protein in varicocele and total population groups. In addition, there was a positive significant correlation between sperm concentration with percentage of DNMT3B positive sperm in varicocele group. Positive significant correlations were also observed between DNMT1 and DNMT3A positive sperm with sperm count in total population and varicocele groups, respectively. In addition, there were positive significant correlations between percentage of motility in DNMT1 positive sperm from each of the three groups. In addition, percentage DNMT3B positive sperm showed a positive significant correlation with sperm motility. Negative correlations were observed between percentage of sperm abnormal morphology with percentage of DNMT1, DNMT3A and DNMT3B positive sperm in varicocele group. Percentage of DNMT3A positive sperm also had negative significant correlation with percentage of sperm abnormal morphology in fertile group. 

### Assessment of localization of DNMTs in the pooled semen samples

We also evaluated percentage of DNMT1, DNMT3A and DNMT3B in over 1000 sperms from five pooled samples from fertile individuals by fluorescence microscope. Percentage of DNMT1, DNMT3A and DNMT3B positive spermatozoa were 71.8%, 57.4% and 38.3%, respectively (Fig .2B). Furthermore, we assessed localization of the three enzymes. The results revealed that localization of DNMT1- positive spermatozoa was mainly in equatorial region (87%). DNMT3B was also mainly localized in equatorial region (almost 53%), but on the head region (almost 35%) of spermatozoa. While DNMT3A was mainly found in the mid-piece region (almost 90%, Fig .2). 

## Discussion

Our findings indicated that in contrast to the percentage of DNMT1 positive sperm, the mean percentages of DNMT3A and DNMT3B positive sperm were significantly higher in varicocele group than fertile group. Similar results were obtained at RNA level. These observations were contradictory to our expectation, since we previously reported lower degree of DNA methylation in spermatozoa of individuals with varicocele (20). Although DNMT1 is known as methyltransferase maintenance, we were unable to observe any significant difference in expression, percentage and intensity of this enzymes between the two groups. This might suggest that altered DNA methylation in varicocele state is not due to aberration of DNA maintenance, but it is rather due to altered activity of DNMT3A and DNMT3B enzymes involved in de novo DNA methylation.

Higher expression of sperm DNMT3A and DNMT3B, both at RNA and protein levels, in individuals with varicocele may reflect aberrant physiological status in testis of these individuals. Considering that presence of RNAs and proteins in sperm reflects spermatogenesis status in testis of corresponding individual (25), one proposition is that cells tried to rectify this abnormal state by increasing activation of DNMT3A and DNMT3B enzymes during spermatogenesis. In addition, high levels of relative fluorescence intensity of DNMT3A and DNMT3B enzymes suggested that not only the percentage of sperm expressing this enzyme, but also amounts of DNMT3A and DNMT3B have significantly increased spermatozoa in infertile men with varicocele, compared to sperm of fertile individuals. In this regard, Hammoud et al. (26) demonstrated increased methylation alteration at six of seven imprinted loci in sperm of men with abnormal protamine content as well as oligozoospermic men.

A second proposition is demethylase activity envisaged for DNMT3A and DNMT3B enzymes in stress condition. Based on the previous study, during oxidative stress, in presence of high calcium and low amount of S-Adenosyl methionine (SAM), these enzymes instead of adding a methyl group to cytosine, they could also function as DNA demethylases or DNA dehydroxymethylases, which are redox state-dependent. Indeed, Ca-dependent apoptosis has been considered as one of the molecular mechanism associated with varicocele. In addition, one carbon cycle-supplementation can improve spermatogenesis in rat varicocele model. Finally, association of varicocele with oxidative stress is well established. Therefore, state of varicocele has three prerequite conditions for DNMTs to act as de-mythylase (21, 27, 28). Therefore, this secondary function displayed by DNMT3A and DNMT3B under oxidative stress conditions like the state of varicocele may physiologically play significant roles in the gene expression via epigenetic regulation. In this state, through DNA demethylation, they may allow genes required to overcome oxidative stress, like antioxidant genes. It is important to note that this proposition remains to be validated at molecular level.

In the state of varicocele due to retarded blood flow and presence of excessive iron precipitation, cells are exposed to oxidative stress and nutritional limitation (21). Therefore, increased expression of DNMT3A and DNMT3B might be related to state of oxidative stress and nutritional limitation in testis of individuals with varicocele. It is also important to note that such role has not been suggested so far for DNMT1 and lack of alteration in expression of this enzyme is consistent with the aforementioned hypothesis. Marques et al. (23) showed that expression of DNMT1 was gradually decreased as spermatogenesis progress, while expression of DNMT3A was decreased from spermatogonia stage to second meiotic division and raised again at round spermatid stage. Expression of DNMT3B is low at spermatogonia stage, increasing primary spermatocytes and decreasing secondary spermatocytes, while it raises again at spermatid stage. Increased expression of these de novo methylation enzymes after meiotic division may suggest their importance in inducing hyper-methylation state in sperm. Hypo-methylation (20) and high expression of DNMT3A and DNMT3B at both mRNA and protein levels may be part of a compensative mechanism of the cell in varicocele state or play a dual role in oxidative stress condition. This observation is consistent with the previous report showing that hypo-methylation is associated with increased expression of DNMT3A and DNMT3B mRNAs in somatic cells. This indicates a compensatory mechanism (29). Recently, Sharma et al. (30) stated that oxidative stress acted as a twin-edged sword in spermatogenesis and it could lead epigenetic deregulation.

Another reason for higher expression of DNMT3A and DNMT3B at mRNA and protein levels in varicocele group compared to fertile group may be due to the reduced expression of chaperone proteins, like heat shock proteins (HSPs). They play an important role in protein folding, which may consequently affect activity of different enzymes (31). This proposition remains to be explored. In this regard, Yesilli et al. (32) showed that varicocelectomy improved expression of HSPA2 and suggested that HSPA2 expression may be considered as a marker of thermal tolerance in men with varicocele.

In this study, percentages of DNMT1, DNMT3A and DNMT3B were assessed in 1000 spermatozoa from fertile individuals by fluorescence microscope. The percentage of DNMTs was within the range of those obtained with flow cytometry. In addition, localization of these DNMTs were assessed in spermatozoa of fertile individuals. DNMT1 and DNMT3A appeared to be mainly localized in equatorial and mid-piece regions, respectively, while DNMT3B mainly localized in equatorial and anterior regions of sperm head. These results were relatively consistent with the images provided by Marques et al. (23); however, they did not carry quantitative analysis. We did not explain for different locations of DNMT3B compared to DNMT1 and DNMT3A, but considering the fact that these DNMTs were mainly localized in equatorial region, and this region contains sperm factors involved in oocyte activation such as Phospholipase C zeta (PLCζ) (33) therefore, we suggest sperm derived DNMT3A and DNM3B could also play a role post sperm - oocyte fusion, like wide DNA de-methylation post fertilization. In this regard, Wang et al. proposed that some of the DNMTs showed differential expression between male and female pronucleus and may account for differences observed in state of methylation between male and female pronuclei (i.e. active vs. passive demethylation). 

Three limitations should be considered for the current study: i. Extraction of RNA was performed in semen samples containing less than one million somatic cell per ml to minimize DNA and somatic cell contamination for the both groups. As shown, the results of relative expression of DNMTs at mRNA were similar to the results obtained from flow cytometry, which only takes into account of the sperm and not the somatic cells. Therefore, we concluded that the significantly higher expression of DNMT3A and DNMT3B at both RNA and protein levels in sperm of individuals with varicocele compared to fertile individuals could not be considered as an artifact. ii. Localization of DNMT1, DNMT3A and DNMT3B enzymes were assessed in over 1000 sperm from five pooled samples of only fertile individuals by fluorescence microscopy, not varicocele group. Further studies are needed to confirm this result. iii. We assessed DNMTs in sperm of infertile men with varicocele and fertile individuals as reflection of testicular function that could not directly be assessed, due to the ethical reasons. 

In this study, we observed significant correlations between percentage of DNMT1 positive sperm with sperm total count, sperm concentration as well as sperm motility, but this correlation was not observed with sperm morphology in total population. Although the correlations were weak, these data indicated that DNMT1 is essential for spermatogenesis and sperm motility. These results were in agreement with previous studies showing that absence of *Dnmts1* was lethal, leading to loss of imprinting, alterations in X chromosome inactivation, genomic instability, increase mitotic recombination rate, chromosome loss and rearrangements (34). In addition, germ line-specific conditional knockdown of *Dnmts1* led to their immediate apoptosis(35); therefore, it is not surprising to see a significant correlation between sperm concentration and sperm DNMT1 level in varicocele and total population. These correlations indicated that reduction in expression of this enzyme in germ cell could lead to apoptosis and cell death, which was consequently related to lower sperm production in varicocele condition. Percentage of sperm DNMT1 level also showed significant correlation with sperm motility in fertile, varicocele and total population groups. This result is in agreement with the previous study performed by Pacheco et al. reporting high prevalence of abnormally methylated CpGs and increase of *DNMT3A* transcript in the low motility samples (36). 

Lack of DNMT3L assessment in our study was based on the previous studies, suggesting that DNMT3L is catalytically inactive and it is mainly expressed in pre-meiotic male germ cells. However, recent studies reported that its absence leads to aberrant expression and methylation of retrotransposons, sever asynapsis and eventual apoptosis of germ cells (37) and it should be considered in future studies. The value of this study would be increased, if we included fertile individuals with varicocele as another group. It is important to note these individuals are not referred to infertility centers and not prepared to provide semen samples, as they are fertile.

## Conclusion

Despite hypo-methylation status of DNA in individuals with varicocele, expression of de novo methylation enzymes were increased in these individuals at both RNA and protein levels. One proposition is that this increase may be related to the dual role played by DNMT3A and DNMT3B enzymes, acting as methyl-transferases in normal conditions as well as dehydroxymethylases in stress conditions, like varicocele. 
